# Risk assessment and antibody responses to SARS-CoV-2 in healthcare workers

**DOI:** 10.3389/fpubh.2023.1164326

**Published:** 2023-07-21

**Authors:** Amit Bansal, Mai-Chi Trieu, Kristin G. I. Mohn, Anders Madsen, Jan Stefan Olofsson, Helene Heitmann Sandnes, Marianne Sævik, Hanne Søyland, Lena Hansen, Therese Bredholt Onyango, Camilla Tøndel, Karl Albert Brokstad, Bergen COVID-19 research group, Håkon Amdam, Heidi Syre, Åse Garløv Riis, Nina Langeland, Rebecca Jane Cox

**Affiliations:** ^1^Department of Clinical Science, Influenza Centre, University of Bergen, Bergen, Norway; ^2^Department of Medicine, Haukeland University Hospital, Bergen, Norway; ^3^Department of Paediatrics, Haukeland University Hospital, Bergen, Norway; ^4^Department of Safety, Chemistry and Biomedical Laboratory Sciences, Western Norway University of Applied Sciences, Bergen, Norway; ^5^Department of Medical Microbiology, Stavanger University Hospital, Stavanger, Norway; ^6^Department of Medicine, Stavanger University Hospital, Stavanger, Norway; ^7^Department of Clinical Science, University of Bergen, Bergen, Norway; ^8^Department of Microbiology, Haukeland University Hospital, Bergen, Norway

**Keywords:** healthcare workers, COVID-19, SARS-CoV-2, spike protein, antibodies, occupational, household

## Abstract

**Background:**

Preventing infection in healthcare workers (HCWs) is crucial for protecting healthcare systems during the COVID-19 pandemic. Here, we investigated the seroepidemiology of SARS-CoV-2 in HCWs in Norway with low-transmission settings.

**Methods:**

From March 2020, we recruited HCWs at four medical centres. We determined infection by SARS-CoV-2 RT-PCR and serological testing and evaluated the association between infection and exposure variables, comparing our findings with global data in a meta-analysis. Anti-spike IgG antibodies were measured after infection and/or vaccination in a longitudinal cohort until June 2021.

**Results:**

We identified a prevalence of 10.5% (95% confidence interval, CI: 8.8–12.3) in 2020 and an incidence rate of 15.0 cases per 100 person-years (95% CI: 12.5–17.8) among 1,214 HCWs with 848 person-years of follow-up time. Following infection, HCWs (*n* = 63) mounted durable anti-spike IgG antibodies with a half-life of 4.3 months since their seropositivity. HCWs infected with SARS-CoV-2 in 2020 (*n* = 46) had higher anti-spike IgG titres than naive HCWs (*n* = 186) throughout the 5 months after vaccination with BNT162b2 and/or ChAdOx1-S COVID-19 vaccines in 2021. In a meta-analysis including 20 studies, the odds ratio (OR) for SARS-CoV-2 seropositivity was significantly higher with household contact (OR 12.6; 95% CI: 4.5–35.1) and occupational exposure (OR 2.2; 95% CI: 1.4–3.2).

**Conclusion:**

We found high and modest risks of SARS-CoV-2 infection with household and occupational exposure, respectively, in HCWs, suggesting the need to strengthen infection prevention strategies within households and medical centres. Infection generated long-lasting antibodies in most HCWs; therefore, we support delaying COVID-19 vaccination in primed HCWs, prioritising the non-infected high-risk HCWs amid vaccine shortage.

## Short summary

Among Norwegian healthcare workers, we found moderate SARS-CoV-2 infection rates (10.5%) in 2020. Infection was associated with household and occupational exposure to SARS-CoV-2. HCWs infected with SARS-CoV-2 had durable antibodies (half-life of 4.3 months) and higher titres post-vaccination than non-infected HCWs.

## Introduction

Understanding the spread of SARS-CoV-2 infection within healthcare facilities and communities is vital to better inform infection prevention and control (IPC) policies. Additionally, identifying the magnitude of risk factors for the coronavirus disease 2019 (COVID-19) in healthcare workers (HCWs) is crucial for healthcare delivery, as these factors may differ between departments, hospitals, regions, and countries. HCWs are at high risk of occupational (1–4) and household (3, 5–8) exposure to SARS-CoV-2; however, existing literature has noted widely varying estimates of rates and risk factors for infection.

Serological testing could complement reverse transcription polymerase chain reaction (RT-PCR) to determine SARS-CoV-2 infection rates over time in low-transmission settings such as Norway (1, 9–11). During the first wave of SARS-CoV-2 in Norway, our preliminary data suggested a low rate of infection by serological testing (1, 12) using orthogonal two-step enzyme-linked immunosorbent assay (ELISA) and neutralisation assays. However, the majority of seroprevalence studies used a single confirmatory serological test (2, 5, 6, 13). Occupational exposure to SARS-CoV-2 and inadequate use of personal protective equipment (PPE) were the main risk factors for SARS-CoV-2 infection among Norwegian HCWs during the first wave (1). A key question is whether SARS-CoV-2 infection rates and risk factors differ during the following COVID-19 waves or the periods between two waves in a low-prevalence setting as different IPC policies were gradually introduced.

Studies have reported conflicting results on the durability of antibodies after mild-to-moderate SARS-CoV-2 infection, either long-term maintenance (up to 1 year) (14–18) or rapid decay (19–22). The evolution of new variants of concern and waning of immunity over time pose a risk for reinfections. Therefore, COVID-19 vaccination is necessary to increase antibody levels in both previously infected (primed) and non-infected (naïve) individuals. In 2021, HCWs were prioritised for the first rounds of COVID-19 vaccination in Norway. HCWs with confirmed SARS-CoV-2 infection in 2020 were recommended one dose of vaccine, while naïve individuals were recommended two doses of vaccine. A few studies have reported higher (23–26) and more durable (25, 26) antibody responses after vaccination in SARS-CoV-2 primed than in naïve individuals. However, since we found higher rates of infection by serological testing than RT-PCR (1), it is still unclear how the magnitude of antibody responses differs after vaccination in HCWs with different pre-existing immunity (24–28).

In this study, we assessed the SARS-CoV-2 infection rates using combined serology and RT-PCR testing, as well as risk factors for infection in HCWs from March to December 2020 in Western Norway, spanning two major regional COVID-19 waves. The main risk factors were further explored and compared to the global data in a meta-analysis. We also assessed the association between prior SARS-CoV-2 infection on antibody levels after the BNT162b2 and/or ChAdOx1-S vaccination in 2021.

## Methods

### Study design

**Figure 1:**
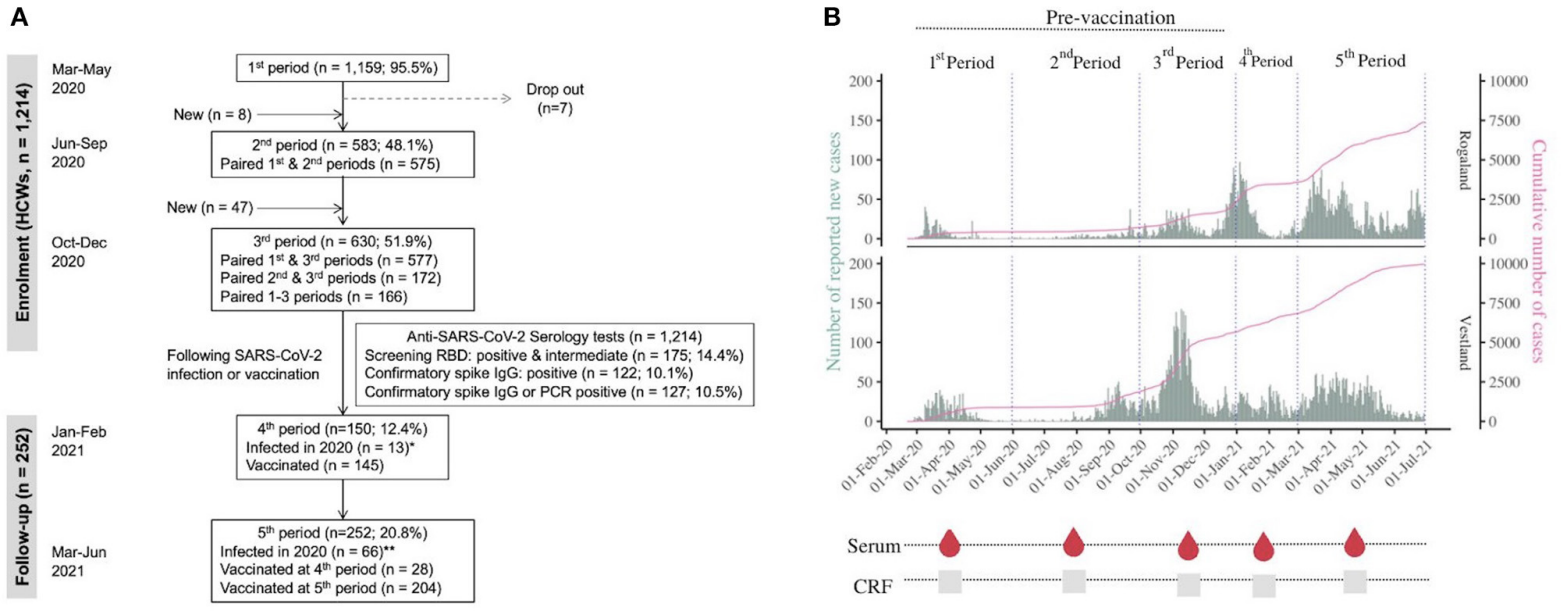
Flow diagram and study procedures. **(A)** Flowchart of study design and healthcare workers (HCWs) selection. HCWs were recruited from the centralised testing centre (Bergen Municipality Emergency covering 284,000 people), Haukeland University Hospital (a university teaching facility and local hospital for ~500,000 people), Haraldsplass Deaconess Hospital (a local teaching hospital providing acute medical care for 145,000 people), and Stavanger University Hospital (teaching hospital providing medical care for 230,000 people). HCWs were recruited from 6 March 2020 before the first hospitalisations on 9th March and the first death on 23rd March and continued up until December 2020, spanning three SARS-CoV-2 periods: March–May 2020 (*n* = 1,159; 95.5%), June–September 2020 (*n* = 583; 48.1%), October–December 2020 (*n* = 630; 51.9%). HCWs with confirmed SARS-CoV-2 infection in 2020 and vaccinated HCWs were invited for follow-up until June 2021. All HCWs included in the immunological analyses provided baseline and follow-up case report forms and serum samples. We performed a two-step orthogonal ELISA testing algorithm. All samples were tested for SARS-CoV-2 receptor-binding domain (RBD)-specific antibodies. Eligible samples were further tested by SARS-CoV-2 anti-spike IgG ELISA for confirmation. *Eight HCWs (or sera samples) from the infected subgroup were also present in the vaccination subgroup; **46 HCWs (or sera samples) from the infected subgroup were also present in the vaccination subgroup. **(B)** Community spread of SARS-CoV-2 virus in Western Norway over time. Daily SARS-CoV-2 positive cases (bars) from the Norwegian Surveillance System for Communicable Diseases (MSIS). The pink line is the cumulative number of deaths. During the study period, anyone who tested positive with a rapid antigen test which was available from December 2020 was encouraged to take a confirmatory RT-PCR test. Results from self-tests were not registered in MSIS. Data on reported cases were therefore not directly comparable over time. Data source (29).

We conducted a prospective cohort study of HCWs in four medical centres in Western Norway, including Bergen Municipality Emergency Room (city testing centre), and three hospitals (Haukeland University Hospital, Haraldsplass Deaconess Hospital, and Stavanger University Hospital). The inclusion criteria were HCWs working during the period 6 March 2020 to 9 December 2020. Exclusion criteria were HCWs who were absent from work due to quarantine or recent RT-PCR-confirmed SARS-CoV-2 infection, therefore posing a risk for active viral shedding and transmission. Serum samples were collected at recruitment and at two follow-up visits during the period from 6 March 2020 to 9 December 2020 (Figure 1A). HCWs with confirmed SARS-CoV-2 infection in 2020 and vaccinated HCWs were invited for follow-up until June 2021. Sera were coded with a unique identification number, aliquoted, and stored at −80°C until use.

### Case report form

A cloud-based case report form was developed using Research Electronic Data Capture (REDCap) (30) to collect relevant clinical and demographic data, such as recent travel history, contact with suspected or confirmed COVID-19 patients, use of PPE, intercurrent illnesses including respiratory disease (fever, dry cough, difficulty breathing, and any other relevant symptoms), and RT-PCR results.

### Vaccine

HCWs were immunized at work in January–March 2021 with either COVID-19 mRNA BNT162b2 (Pfizer/BioNTech) or viral vector-based ChAdOx1-S vaccine (Oxford/AstraZeneca), encoding the spike protein of SARS-CoV-2 Wuhan-Hu1 strain (NCBI, NC_045512). A second dose was provided 21–84 days after the initial dose according to national guidelines for prioritisation, which is based on criteria, such as age, occupation, and comorbidities. HCWs who received the first dose of the ChAdOx1-S vaccine subsequently received the BNT162b2 vaccine as the second dose, due to a suspected link between the ChAdOx1-S vaccine and some rare but serious events (thrombosis and thrombocytopenia) (31).

### Antigens

The SARS-CoV-2 receptor-binding domain (RBD) and full-length spike proteins were produced and purified in-house as previously described (32).

### Enzyme-linked immunosorbent assay

Sera were heat-inactivated for 1 h at 56°C before testing in a two-step orthogonal ELISA (1, 10, 12, 32, 33): screening for detection of RBD-reactive samples followed by a confirmatory spike protein ELISA, as previously described (1, 10, 32). Briefly, sera with positive or intermediate optical density (OD) values for RBD (OD >0.430) in screening were serially diluted and tested in the anti-spike IgG ELISA (1). IgG endpoint titres were calculated as the reciprocal of the serum dilution giving an OD value of three standard deviations above the mean of historical pre-pandemic sera (*n* = 128) using Prism version 8.4.2 (GraphPad). IgG endpoint titres ≥100 were considered positive.

### Statistics

HCW characteristics were stratified by risk factors and infection status across the periods of different SARS-CoV-2 infectivity. Statistical analyses were performed in R version 4.2.2 for macOS using lme4, meta, ggplot2, ggeffects, ggthemes, patchwork, mgcv, epiR, and performance packages. The Clopper–Pearson interval or exact method was used to estimate cumulative probabilities of the binomial distribution. The function epi.conf of the package epiR was used to compute the mean and 95% confidence intervals for the prevalence and incidence rates. We censored person-time when HCWs withdrew from the study. Comparisons between HCWs were made based on demographic, clinical characteristics, exposure factors, and serological data or infection status using univariate models (Kruskal–Wallis rank sum test or Pearson's chi-squared test, as appropriate). Variables of interest were time (days since the seropositive SARS-CoV-2 diagnosis or first dose of vaccination), age, sex, occupational and household exposure to patients with COVID-19, travel history, study site (categorised as Bergen or Stavanger), occupation, prior infection/vaccination against SARS-CoV-2, clinical symptoms, and comorbidities (hypertension, diabetes, and heart disease). Generalised additive mixed model including population-level fixed effects and individual random effects was used to calculate adjusted infection rates and adjusted odd ratios (aORs). The generalised additive model was fitted using the gam function (of the mgcv package) with random effects smooth term or penalized regression splines by a ridge penalty (i.e., the identity matrix). Antibody dynamics were plotted by linear mixed-effects exponential decay models, fitted by the lmer function of the lme4 package. The yielding linear equation of the model was log(*y*) = log(α) + log(β) ^*^ time = A + B ^*^ time since seropositivity. We performed mixed-effects models to determine the effects of infection and/or vaccination against SARS-CoV-2 on antibody responses with adjustment for relevant demographic factors and individual random effects, using the restricted maximum likelihood (REML) approach. All tests were two-sided, and *p*-values < 0.05 were considered statistically significant. The figures were made using PowerPoint version 16.69.1, R version 4.2.2, BioRender.com, and Canva version 1.56.0.

### Meta-analysis

To compare the magnitude of risk factors for seropositivity to SARS-CoV-2 in our Norwegian HCW data with existing global literature, we searched the electronic databases (MEDLINE, CINAHL, Google Scholar, and EMBASE) and meta-analysis was performed (search strategy, Supplementary Figure 1).

#### Eligibility criteria

Eligible studies met the following inclusion criteria: (1) published from 1 January 2020 to 31 December 2022 and (2) evaluated the association between occupation and/or household exposure to SARS-CoV-2 and anti-SARS-CoV-2 IgG seropositivity.

#### Selection and screening of articles

Original research articles published in English during the COVID-19 pandemic until 31 December 2022 were searched for prospective and retrospective full-text studies that reported quantitative data on the association between SARS-CoV-2 spike-specific IgG antibodies in HCWs and occupational exposure (low- or high-risk groups; treating patients with or without PPE) or household exposure. Articles resulting from these searches and relevant references cited in those articles were reviewed. A total of 4,544 studies were assessed. We excluded 820 studies due to duplicates and 3,599 studies deemed ineligible based on the title and abstract. Of the remaining 125 studies, 20 studies met the selection criteria including the current Norwegian cohort study. The ORs with a 95% confidence interval (CI) from individual studies were calculated as described in the Supplementary material.

The Mantel–Haenszel method was used for the pooling of studies under the fixed-effects model, with random effects variants for the calculation of the between-study heterogeneity variance using the REML method (i.e., calculating the weights) for using metabin function (meta package) in R.

### Patient consent statement

The Western Norway Ethics Committee approved the study (No. 118664 and 218629). All HCWs provided written informed consent before inclusion.

## Results

### Study population: Norwegian HCW cohort

From the start of the COVID-19 pandemic in February 2020, cases were well-defined in Western Norway due to early centralised SARS-CoV-2 RT-PCR testing. In this study, we commenced recruitment of HCWs from 6 March 2020 before the first COVID-19 hospitalisations and deaths were reported in the Western Norway region (1, 29) and until December 2020 (Figure 1A). The study was conducted in a low community transmission setting, spanning two major regional COVID-19 waves in Western Norway (Figure 1B). HCWs (*n* = 1,214), contributing to 848 person-years of follow-up time, were enrolled from the main medical centres of Western Norway, including 505 nurses (42%) and 265 physicians (22%). The median age was 40 years (range 19–78 years), and 80% of HCWs were female. Full descriptive analyses of the demographic and clinical characteristics of HCWs are provided in Table 1 and Supplementary Tables 1–5. Only two HCWs were over 70 years old. HCWs were categorised by their occupational exposure: high-risk exposure to SARS-CoV-2 (*n* = 729, 60%) and low-risk (*n* = 485, 40%) with no or minimal occupational exposure to SARS-CoV-2. International travel history was recorded in 7.7% (74/962) of HCWs. COVID-19-like symptoms were reported at recruitment in only 181/1,159 (15.6%) of HCWs (Supplementary Table 1), of whom 30 HCWs tested positive for SARS-CoV-2 by RT-PCR.

**Table 1:** Characteristics of healthcare workers enrolled in the cohort study in 2020.

**Characteristic**	**HCWs recruited during 2020 (*N* = 1,214)^a^**	**Not-infected^b^ with SARS-CoV-2 (*n* = 1,087)^a^**	**Infected^b^with SARS-CoV-2 (*n* = 127)^a^**	***p*-value^c^**	**Adjusted odds ratio (95% CI)^d^**	**Adjusted *p*-value^d^**
Age (year): median (IQR)	40 (30–51)	39 (30–51)	41 (30–50)	0.8	1.0 (1.0–1.1)	0.8
Sex: female	967/1,202 (80%)	867/1,077 (81%)	100/125 (80%)	0.9	0.4 (0.1–1.1)	0.071
**Occupational exposure** ^e^						
Low	485/1,214 (40%)	455/1,087 (42%)	30/127 (24%)	**< 0.001**	Ref.	0.4
High	729/1,214 (60%)	632/1,087 (58%)	97/127 (76%)		1.6 (0.5–4.7)	
**Household exposure**	107/1,161 (9.2%)	88/1,076 (8.2%)	19/85 (22%)	**< 0.001**	8.8 (2.4–32.1)	**0.001**
**Travel history**						
Domestic	631/962 (66%)	587/882 (67%)	44/80 (55%)	**0.042**	–	0.1
International	74/962 (7.7%)	63/882 (7.1%)	11/80 (14%)		2.9 (0.7–12.0)	
No	257/962 (27%)	232/882 (26%)	25/80 (31%)		Ref.	
**Study site**						
Bergen	858/1,214 (71%)	764/1,087 (70%)	94/127 (74%)	0.4	Ref.	**0.030**
Stavanger	356/1,214 (29%)	323/1,087 (30%)	33/127 (26%)		0.2 (0.1–0.9)	
**Occupation**						
Physician	265/1,214 (21.8%)	247/1,087 (22.7%)	18/127(14.2%)	0.4	0.5 (0.1–2.2)	0.4
Nurse	505/1,214 (41.6%)	462/1,087 (42.5%)	43/127 (33.9%)		0.6 (0.2–1.9)	
Other	444/1,214 (36.6%)	378/1,087 (34.8%)	66/127 (52%)		Ref.	
**Comorbidity^f^**	101/1,154 (8.8%)	94/1,072 (8.8%)	7/82 (8.5%)	0.9	0.3 (0.1–2.3)	0.3

a Data are number (%) except for age, showing the median age (years old) with interquartile range (IQR).

b Infection status was determined by SARS-CoV-2 serology and/or RT-PCR testing.

c Characteristics of infected and non-infected HCWs were compared in Kruskal-Wallis rank sum test or Pearson's Chi-squared test as appropriate. p-values < 0.05 are considered statistically significant and in bold.

d Generalised additive mixed model including population level fixed effects and individual random effects was used to calculate adjusted odd ratios and 95% confidence interval (CI). Due to low community spread in Norway, we did not find domestic travel as a risk factor.

e High-risk occupational group that tested and treated COVID-19 patients includes ambulances services; emergency, infectious diseases, anesthesia, and intensive care unit departments at Haukeland University Hospital, Haraldsplass Deaconess Hospital, Bergen Municipality Emergency Room, and Stavanger University Hospital (SUH). Maternity ward of women's clinic at SUS was also deemed to be high-risk. Low-risk group that did not treat COVID-19 patients includes other clinical departments and laboratories.

f Comorbidities include hypertension, diabetes and heart diseases.

### Moderate SARS-CoV-2 infection rates among Norwegian HCWs in 2020

**Figure 2:**
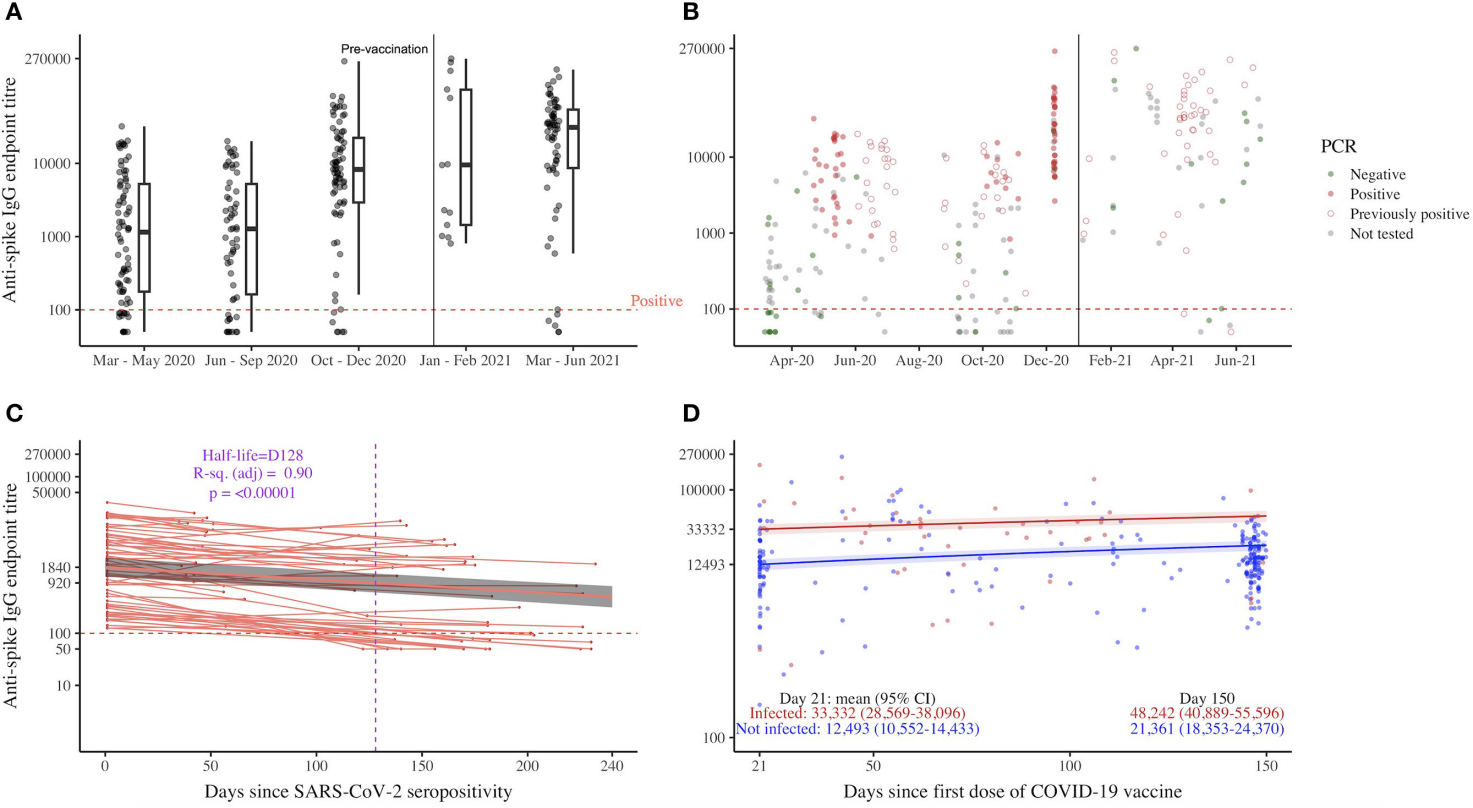
SARS-CoV-2 spike-specific IgG antibodies in healthcare workers, HCWs. Each circle/symbol represents one individual HCW. IgG, immunoglobulin G; SARS-CoV-2, severe acute respiratory syndrome coronavirus 2. **(A, B)** Spike-specific IgG endpoint titre transformed on a log_10_-based scale (*y*-axis) and month-year format (*x*-axis), *n* = 122. A vertical line divides the time prior to vaccines were available and when vaccination was recommended. The horizontal dash line shows the cut-off of the spike IgG ELISA. **(B)** RT-PCR results are color coded with dark green for negative and firebrick red color for positive test results. **(C)** The data were collected in 2020 (prior to COVID-19 vaccination), and samples were repeated over time (*n* = 63). The mixed-effects model with exponential decay was used to analyse antibody waning. The model included population-level fixed effects and individual random effects and fit using the lmer function (lme4 package) in R version 4.2.2. Antibodies were naturally log transformed. The trend line, back-transformed estimated mean (red), is smoothed across the 95% confidence interval values (gray shade). **(D)** Spike-specific IgG endpoint titre transformed (*y*-axis) with samples repeated over time and days since the first dose of the COVID-19 vaccine (*x*-axis). The mixed-effects models were performed with population-level fixed effects and individual random effects and fit using the lmer function (lme4 package) in R version 4.2.2. The trend line, back-transformed estimated mean, is smoothed across the 95% confidence interval values.

We used orthogonal serological testing to confirm SARS-CoV-2 infection. HCWs who were intermediate or positive for RBD and subsequently positive for anti-spike IgG (Figure 2A) were considered sero-confirmed infections. In total, we identified 122/1,214 HCWs infected with SARS-CoV-2 by serological testing throughout 2020, which was equivalent to a prevalence of 10.0% (95% CI: 8.4–11.9) and an incidence rate of 14.4 cases per 100 person-years (95% CI: 12.0–17.2). By combining serological and RT-PCR testing, a total of 127 HCWs SARS-CoV-2 infections were identified, although not all HCWs were tested by RT-PCR (Figure 2B). The overall prevalence was 10.5% (95% CI: 8.8–12.3), and the incidence rate was 15.0 cases per 100 person-years (95% CI: 12.5–17.8). During the three study periods in 2020, the seropositivity rates gradually increased from 5.7% (66/1,159) in March–May (the first COVID-19 wave) to 7.9% (46/583) in June–September, and the highest 11.3% (71/630) in October–December (the second wave; Table 2). We identified 5 (0.9%) and 51 (8.4%) new SARS-CoV-2 seropositive cases during the second and third periods, respectively. We found higher positivity rates by serological testing than by RT-PCR, and 17 HCWs were RT-PCR-negative but seropositive (Table 2 and Figure 2B), suggesting that serological testing was more sensitive for surveillance than RT-PCR.

**Table 2:** SARS-CoV-2 infection rates among healthcare workers in Western Norway during March–December 2020.

**Variable**	**1st period (March–May) *N* = 1,159^a^**	**2nd period (June–Sep) *N* = 583^a^**	**3rd period (October–December) *N* = 630^a^**	**2020 (March–December) *N* = 1,214^a^**	**Prevalence per 100 HCWs in 2020 (95% CI)^b^**	**Incidence rate in cases per 100 person-years (95% CI)^b^**
Seropositive for anti-spike IgG^c^	66 (5.7%)	46 (7.9%)	71 (11.3%)	122 (10.0%)	10.0 (8.4–11.9)	14.4 (12.0–17.2)
New seropositive cases	66 (5.7%)	5/542 (0.9%)	51/610 (8.4%)	–	–	–
RT-PCR positive	30/223 (13.5%)	4/94 (4.3%)	49/213 (23.0%)	52/301 (17.3%)	4.3 (3.2–5.6)	6.1 (4.6–8.1)
New positive cases by serology and/or RT-PCR	66 (5.7%)	8/542 (1.5%)	53/610 (8.7%)	–	–	–
Total positivity by serology and/or RT-PCR	66 (5.7%)	49 (8.4%)	73 (11.6%)	127 (10.5%)	10.5 (8.8–12.3)	15.0 (12.5–17.8)

a n/N (%).

b Clopper-Pearson interval or exact method was used to estimate cumulative probabilities of the binomial distribution in a population of 33,996 employed HCWs in Bergen and Stavanger in health care and social services with healthcare education (34, 35). For incidence rate calculation, a total of 122 (serology) or 52 (RT-PCR) or 127 (serology and/or RT-PCR) SARS-CoV-2 infection diagnoses were made from 847.74 person-years at risk.

c Sera were confirmed in an anti-spike IgG ELISA after screening anti-RBD Ig. HCWs were 114 (9.8%), 53 (9.1%), and 71 (11.3%) during the 1–3 periods, respectively, and 175 (14.4%) during March–December 2020.

–, not applicable; CI, confidence interval; HCWs, Healthcare workers; RT-PCR, reverse transcription polymerase chain reaction.

### Risk factors for infection: high household and modest occupational risk of SARS-CoV-2 infection among Norwegian HCWs in 2020

We found that the SARS-CoV-2 infection rate (confirmed by seropositivity and/or RT-PCR) was significantly higher in HCWs with occupational and household exposure to the virus (*p* < 0.001), as well as an international travel history (*p* = 0.04; Table 1) by univariate analysis, although there were no significant differences by age, sex, and comorbidities (*p* > 0.05). Separate analyses of the three study periods in 2020 showed that SARS-CoV-2 infection was significantly associated with household exposure (*p* < 0.001) during March–May (the first COVID-19 wave), with occupational exposure (*p* < 0.001) during October–December (the second wave), while only international travel history (*p* = 0.005) was a significant risk factor for infection during June–September (a period of very low community prevalence between the two waves; Supplementary Table 1).

In a generalised additive mixed-effects model, HCWs with household exposure to SARS-CoV-2 had significantly higher odds of being infected than those with no exposure (aOR 8.8, 95% CI: 2.4–32.1, *p* = 0.001; Table 1, Supplementary Table 2). The odds of infection were higher in HCWs with international travel history (aOR 2.9, 95% CI: 0.7–12.0, *p* = 0.1) and occupational exposure (aOR 1.6, 95% CI: 0.5–4.7, *p* = 0.4), albeit not statistically significant. Regionally, we found higher infection rates in the larger city (Bergen 22%, 95%CI: 8–48 vs. Stavanger 6%, 95%CI: 1–23; *p* = 0.03), although there was uniform IPC policy between our study sites (Table 1, Supplementary Tables 1–3).

### Meta-analysis of risk factors for infection: high household and modest occupational risk of SARS-CoV-2 infection among HCWs globally in 2020

**Figure 3:**
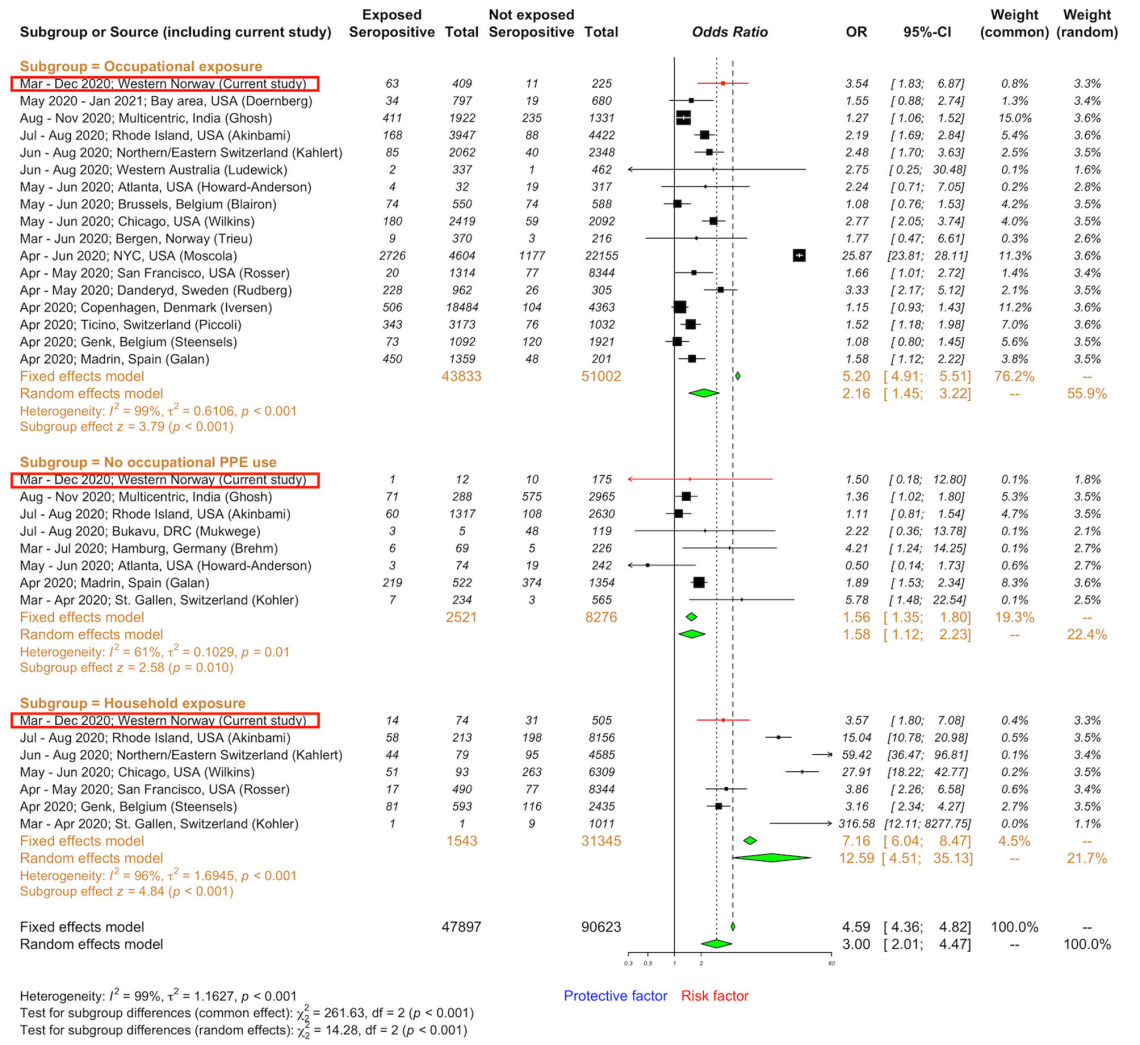
Forest plot to evaluate whether SARS-CoV-2 spike-specific IgG seropositivity rates differed with occupational exposure and household exposure among healthcare workers. Data are compiled from a meta-analysis of literature (Supplementary Figure 1) and include our data presented as odds ratios and 95% confidence interval, CI. Subgroups: occupational (1–6, 8, 13, 36–43) exposure to SARS-CoV-2 cases, no PPE use at work (5, 7, 13, 38, 43–45), and household (5–7, 39, 42) exposure to SARS-CoV-2 cases. Meta-analysis of effect estimates was performed using the metabin function (meta package) (46) in R version 4.2.2.

We further explored the risk factors for SARS-CoV-2 IgG seropositivity [occupational exposure to SARS-CoV-2 (1–6, 8, 13, 36–43), no PPE use at work (5, 7, 13, 38, 43–45), and household exposure to SARS-CoV-2 (5–7, 39, 42)] comparing our data to existing literature (Figure 3 and Supplementary Figure 1). Global data from 20 studies, including 138,520 HCWs, were analyzed. We extracted the number of HCWs in SARS-CoV-2 exposed and unexposed groups that developed SARS-CoV-2 IgG seropositivity from individual studies and incorporated corresponding data from our study to calculate crude ORs. Compared to our risk assessment data in Norwegian HCWs (occupational exposure OR 3.5, 95% CI: 1.8–6.9; occupational exposure without PPE use OR 1.5, 95% CI: 0.2–12.8; household exposure OR 3.6, 95% CI: 1.8–7.1), the global pooled ORs for SARS-CoV-2 IgG seropositivity were lower for occupational exposure (OR 2.2; 95% CI: 1.4–3.2), similar for occupational exposure without PPE use (OR 1.6; 95% CI: 1.1–2.2), but much higher for household exposure (OR 12.6; 95% CI: 4.5–35.1).

### Durable antibodies after SARS-CoV-2 infection and vaccination among Norwegian HCWs in 2020–2021

Binding antibodies to the SARS-CoV-2 full-length spike protein were assessed in HCWs. The participant-level temporal sequence of testing results for all seropositive HCWs in 2020 (*n* = 122) is depicted in Supplementary Figure 2. We evaluated the durability of antibody responses after SARS-CoV-2 infection in a longitudinal cohort with paired samples (*n* = 65) before the start of COVID-19 vaccination. Two HCWs had antibody titre increases of 4.4- and 9.7-fold within 3.5 and 7.3 months since their seropositivity, respectively, probably due to reinfection, and therefore were excluded in further analysis. A mixed-effects model was fitted for anti-spike IgG endpoint titres against days since seropositivity in 63 remaining HCWs (Figure 2C). We found that antibodies gradually waned over time with a half-life of 4.3 months (mean titre 920; 95% CI: 604–1,397) from the time of seropositivity and stayed above the cutoff for seropositivity at 8 months (mean titre 500; 95% CI: 313–801). Only 15 HCWs (23.8%) became seronegative by 8 months. No significant difference in demographic or clinical characteristics was found between HCWs who maintained their seropositivity by 8 months (sustainers, *n* = 48) and those who did not (*n* = 15; Supplementary Table 4). As expected, sustainers had higher baseline binding antibodies to the SARS-CoV-2 RBD and full-length spike proteins (i.e., on day 1 of the seropositive test result) than HCWs who became seronegative with antibodies waning below the detection level.

We continued to prospectively follow up HCWs who received COVID-19 vaccination and volunteered for continued follow-up until June 2021 (Figure 1, Supplementary Table 5). Of 232 vaccinees, 46 (19.8%) HCWs tested positive and 186 (80.2%) HCWs tested negative for SARS-CoV-2 by serology and/or RT-PCR in 2020. All HCWs developed durable spike-specific IgG antibodies 5 months after the first dose of the COVID-19 vaccine (Figure 2D). However, HCWs who were previously infected with SARS-CoV-2 had higher spike-specific IgG antibody titres after vaccination (mean titre at 5 months 48,242; 95% CI: 40,889–55,596) than naïve HCWs (mean 21,361; 95% CI: 18,353–24,370).

## Discussion

**Figure 4:**
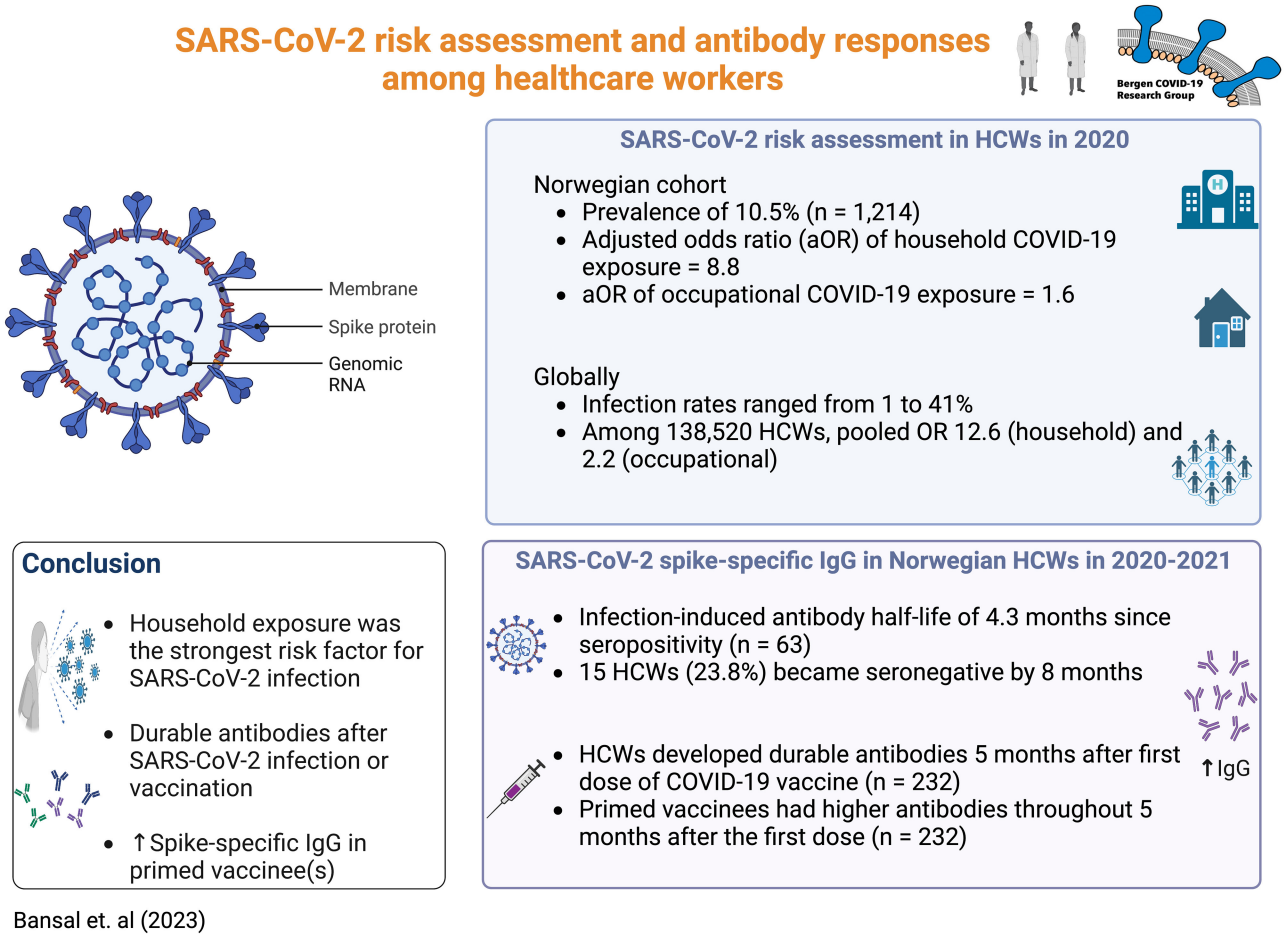
Summary of key findings. Created with BioRender.com (2023).

During the COVID-19 pandemic, HCWs have globally experienced considerable morbidity and mortality from SARS-CoV-2. The infection and mortality rates differ widely (3, 8, 44) depending on the levels of community spread, IPC policies, and availability of PPE. In this large cohort observational study, we identified a moderate SARS-CoV-2 infection rate (10.5%) in Norwegian HCWs in 2020. Studies have reported similar SARS-CoV-2 infection rates [5%−14% in Belgium (8), Spain (47), Switzerland (37), Canada (48), USA (4, 5, 13, 39), and Australia (49)], while others have reported substantially lower [ ≤ 4% in Denmark (2), Germany (50), Greece (51), Switzerland (7, 42), USA (3, 6), Australia (41, 52, 53), India (54), Japan (55, 56)] or higher [16%−41% in Germany (57), Spain (38), Sweden (36), India (43, 58), Democratic Republic of Congo (44)] infection rates than our study (Figure 4). Higher SARS-CoV-2 seroprevalence in HCWs than in the community has been described [Greece (51) and Sweden (36)]. In agreement, the regional community infection rate in 2020 was estimated at < 4%, using the national SARS-CoV-2 RT-PCR or seroprevalence Norwegian data (9, 11), and is lower than the 10.5% infection rate in HCWs found in this study. Importantly, our findings were based on both RT-PCR and serological results of longitudinal samples from 1,214 HCWs recruited from four main medical centres in two different major cities of Western Norway, making this study the largest serological survey representative of the region.

We conducted a meta-analysis of global published data on risk factors related to SARS-CoV-2 infection in HCWs and found that the pooled crude OR for IgG seropositivity in HCWs was greater with higher occupational and household exposures and while managing patients without PPE (Figure 3, Supplementary Figure 1). In our cohort of Norwegian HCWs, SARS-CoV-2 infection was associated with household contact during the first COVID-19 wave, occupational exposure during the second wave, and international travel history during the periods between the two waves. However, only household contacts remained a statistically significant risk factor in the generalised additive mixed-effects model (aOR 8.8, 95% CI: 2.4–32.1; Table 1). Compared to other settings (e.g., at work), households might use IPC recommendations less frequently. Our findings do not establish causation. Spousal/partner contact, household crowding (e.g., number of people in the house and per room), and commonly touched surfaces are likely contributors to SARS-CoV-2 transmission, although this was not directly assessed.

In March 2020, imported SARS-CoV-2 cases and local spread increased rapidly in Norway, and the first mortality was reported on 13 March 2020 (29). A national shutdown on 12 March 2020 reduced community transmission, and the epidemic curve started to decline on 24 March 2020 (1). Community transmission remained low (11) (Figure 1B) as reflected in our 2020 data. At the start of the COVID-19 pandemic, there were local reports of PPE shortage in the community (nursing homes and home services) (59). However, the four medical centres in our study adopted an easy-to-follow IPC policy against any respiratory infection, focusing on both occupational and household exposure, ensuring adequate staff training, and prioritised PPE for HCWs with higher exposure to SARS-CoV-2 (Supplementary Tables 3, 6). Quarantine of close contacts can further reduce ongoing SARS-CoV-2 transmission in low-transmission settings (60). The Norwegian government invested in convenient RT-PCR testing and vaccination schedules, statutory sick leave to quarantine HCWs due to infection or exposure to household members with SARS-CoV-2, and publishing frequent public health advice (Supplementary Tables 7, 8). This probably helped to reduce the risk of infection due to occupational exposure in our Norwegian HCWs throughout 2020, while household exposure to SARS-CoV-2 is likely a major risk for infection in HCWs as shown in our data and existing literature (8, 39, 42). Hence, IPC policies for HCWs should include advice after respiratory viral exposure at both work and home.

To strengthen IPC policies, it is critical to know whether HCWs develop symptoms and long-term immune memory against SARS-CoV-2. The majority of seropositive HCWs identified in our study had no symptoms (53/80, 66%), and no HCW was admitted to intensive care or died due to COVID-19, indicating that they had the mild-to-moderate infection. Such infection induced durable anti-spike IgG antibodies with a half-life of 4.3 months after the first seropositive test result. The majority (48/63, 76%) of HCWs remained seropositive for at least 8 months. This finding is consistent with others reporting antibody maintenance for up to 12 months post-infection (14–18). However, rapid decaying antibodies after mild SARS-CoV-2 infection (19–22), especially weaker immune responses in asymptomatic individuals (20), have also been reported. The spike-specific binding antibodies are likely secreted from plasma cells which are promptly generated from B cells, after their activation with the SARS-CoV-2 RBD and spike proteins. These antibodies may interfere with the binding of SARS-CoV-2 to the human cell angiotensin-converting enzyme 2 (ACE-2) receptor and may, in theory, neutralise the SARS-CoV-2 virus and prevent reinfection. A decline in the circulating RBD and spike antibodies is an expected finding and is probably due to the disappearance of the short-lived immature plasma cells or plasmablasts. Estimating and predicting the durability of antibodies from natural infection is more crucial as mitigation strategies, such as mask use and physical distancing, have become more relaxed and more variants of concern may continue to appear. In our study, 24% (15/63) of infected HCWs became seronegative by 8 months, suggesting a need for COVID-19 vaccination in HCWs ~6 months after mild-to-moderate SARS-CoV-2 infection (61). Furthermore, the humoral response to SARS-CoV-2 after infection and/or vaccination is likely heterogeneous (24–28, 62). A history of SARS-CoV-2 infection would have influenced the immune response to COVID-19 vaccination in vaccinees. Our previous study showed that one dose of COVID-19 vaccination induced robust antibody responses in naïve vaccinees (33) and others reported that SARS-CoV-2 primed vaccinees mount significantly higher antibodies after vaccination than naïve individuals (23–26, 63). In agreement, we also found that spike-specific IgG antibody titres were consistently higher in HCWs with prior SARS-CoV-2 infection than naïve HCWs throughout 5 months after the first COVID-19 vaccination, consequently giving the primed HCWs an added benefit of circa 2.3 times higher antibody titres at 5 months after the first dose (Figure 2D). Thus, prior SARS-CoV-2 infection should be taken into consideration for the tailored deployment of vaccination regimes, such as delaying vaccination in previously infected individuals for up to 5–6 months after infection to prioritise the naïve high-risk groups in the event of a vaccine shortage.

Key strengths of our study are the inclusion of a large cohort of HCWs with longitudinal combined serological and RT-PCR follow-up and in-depth analysis of risk factors and humoral immune responses. Studies using either serological or RT-PCR testing on single time-point samples may result in biased estimates of the true infection rates (3, 50). A low-infection rate of 1.5% was reported in a Norwegian RT-PCR-based register study in week 9 of 2020 (64). Furthermore, asymptomatic HCWs may not get tested by SARS-CoV-2 RT-PCR, which would underestimate the total number of infections. This is particularly important because the majority of HCWs with SARS-CoV-2 infection (25%−62% over three periods) in our study were asymptomatic in 2020 (Supplementary Table 1), agreeing with previous reports of asymptomatic infection in approximately half of HCWs (3, 38, 47, 56). By combining frequent longitudinal serological and RT-PCR testing, the infection rates and risk factors among HCWs can be more correctly calculated. The change in the infection rates among HCWs over three study periods in 2020 mirrored the community spreads, in which higher infection rates in HCWs were found during the COVID-19 waves, suggesting that infection was not necessarily acquired in healthcare settings.

Our study relied on self-reporting questionnaires for assessing household exposure, travel, and vaccination history; hence, recall bias is an expected limitation. However, the high risk of occupational exposure was determined by the working department and contact with patients having COVID-19 rather than only subjective exposure experiences. In fact, we did not find an association between subjective occupational exposure experiences and SARS-CoV-2 infection. The SARS-CoV-2 RT-PCR tests were conducted based on national criteria (unprotected patient or household exposure to SARS-CoV-2, travel history, and clinical symptoms); therefore, RT-PCR results alone should be inferred with caution as the majority of our HCWs infected with SARS-CoV-2 were asymptomatic. Although we had a uniform IPC policy and national advice, the pandemic waves and infection rates during the Autumn 2020 slightly differed between the two cities studied (Figure 1B, Table 1). We found lower infection rates in the smaller city (Stavanger, Rogaland). The national data estimated lower COVID-19-related mortality in Rogaland than in Vestland (11 vs. 67 deaths; 2.3 vs. 10.5 per 100,000 inhabitants) (29), which agreed with our findings. Furthermore, HCWs were recommended a second vaccine dose between 21 and 84 days after the first dose; thus, it is likely that many vaccinees in our study had received a second dose, which explain the slightly higher antibody titres at day 150 compared to day 21 after the first dose (Figure 2D). The data collection in Norway began when pre-alpha lineages were predominant and continued until the delta variant took over (March 2020–June 2021) in Norway. Therefore, our results cannot be directly extrapolated to the more recent SARS-CoV-2 variants. Nevertheless, they are still highly relevant for hospitals and medical settings where SARS-CoV-2 infection rates are low in the community.

During the COVID-19 pandemic, our data highlight the importance of serological tests to supplement rapid and frequent RT-PCR testing of HCWs and tailored vaccine deployment after natural immunity to strengthen evidence-based pandemic preparedness. COVID-19 vaccination is required to protect against reinfection in previously infected individuals. However, our findings suggest that vaccination schedule can be delayed up to 5–6 months post-infection to prioritise naïve high-risk groups during a vaccine shortage, as infection induced long-lasting antibodies and primed vaccinees had higher titres than naïve vaccinees throughout 5 months post-vaccination. Globally, we advocate adherence to isolation and precautions against SARS-CoV-2 exposure, not only at work but also at home. More research studies should be carried out on preventive measures such as transmission modes and mask use at home or work, ventilation systems at work, and the role of statutory sick leave and voluntary isolation at external facilities.

## Data availability statement

The datasets presented in this article are not readily available because data is not released due to General Data Protection Regulation (EU GDPR). Small subgroups of healthcare workers make the risk of identification of sensitive data of individual healthcare worker possible; therefore, the data are not openly accessible. Requests to access the datasets should be directed at: RC, rebecca.cox@uib.no.

## Ethics statement

The studies involving human participants were reviewed and approved by the Western Norway Ethics Committee approved the study (No. 118664 and 218629). All HCWs provided written informed consent before inclusion. The participants provided their written informed consent to participate in this study.

## Bergen COVID-19 research group

Håkon Amdam, Geir Bredholt, Nina Urke Ertesvåg, Elisabeth Berg Fjellveit, Sarah Lartey, Fredrik Grøvan, Hauke Bartsch, Kanika Kuwelker, Juha Vahokoski, Bård Kittang, Dagrun Waag Linchausen, Bjørn Blomberg, Fan Zhou.

## Author contributions

RC, NL, KB, KM, and CT conceptualized and organized the study. AB, HSa, MS, HSø, ÅR, KM, JO, M-CT, and TO collected case report forms and/or sera. AB, AM, JO, TO, LH, and M-CT performed experiments. AB visualized the data, performed statistical and meta-analyses, and wrote the original draft of the manuscript. All authors reviewed and amended the draft.
