# Supplementary material for: Risk assessment and antibody responses to SARS-CoV-2 in healthcare workers
Source: Front Public Health. 2023 Jul 21;11:1164326. doi: 10.3389/fpubh.2023.1164326 (PMC10402899; doi:10.3389/fpubh.2023.1164326)
Supplement: Supplementary file 1 [file Data_Sheet_1.pdf]

Supplementary methods

Meta-analysis
To compare the magnitude of risk factors for seropositivity to SARS-CoV-2 in our Norwegian HCW data with existing global literature, we searched the electronic databases (MEDLINE, CINAHL, Google Scholar and EMBASE) and meta-analysis was performed (search strategy, Supplementary Figure 1).

Eligibility criteria: Eligible studies met the following inclusion criteria: 1) published from January 1, 2020, to December 31, 2022; and 2) evaluated the association between occupational and/or household exposure to SARS-CoV-2 and risk of anti-SARS-CoV-2 IgG seropositivity.

Selection and screening of articles*:* Original research articles published in English during the COVID-19 pandemic until 31 December 2022 were searched for prospective and retrospective full-text studies that reported quantitative data on association between SARS-CoV-2 spike-specific IgG antibodies in HCWs and occupational exposure (low or high-risk groups; treating patients with or without PPE) or household exposure. Articles resulting from these searches and relevant references cited in those articles were reviewed.

A total of 4,544 studies were assessed. We excluded 820 studies due to duplicates and 3,599 studies deemed ineligible based on the title and abstract. Of the remaining 125 studies, 20 studies met the selection criteria including the current Norwegian cohort study. The ORs with 95% confidence interval (CI) from individual studies was calculated as described here:

		EXPOSURE: Occupational or household exposure to SARS-CoV-2 viruses	
		Exposed	Non-Exposed
OUTCOME: SARS-CoV-2 infection	Seropositive:	a	b
	Seronegative:	c	d

OR = (ad)/(bc)

The Mantel-Haenszel method was used for pooling of studies under the fixed effect model, with random effects variants for the calculation of the between-study heterogeneity variance using REML method (i.e., calculating the weights) using metabin function [meta package(1)] in R.
Both common effect and random effects models were plotted. Argument subgroup was used to conduct subgroup analysis for a categorical viral exposure covariate.
OR (Mantel-Haenszel method) = $\Sigma(a_i d_i/n_i)/ \Sigma(b_i c_i/n_i)$
- where $n_i = a_i+b_i+c_i+d_i$.

The I^2 and tau-squared ($\tau 2$ or Tau2) statistic were used to see if the studies chosen were different from each other (i.e., between-study variance). To be precise, the I^2 number measures percentage of total variability due to heterogeneity between studies, rather than just by chance. Substantial percentage of total variability due to between-study heterogeneity was found (I^2= 99%). Furthermore, we found asymmetrical funnel plot, which suggest either publication bias, or more benign reasons such as between-study heterogeneity or small number of studies between subgroups, or by chance. The Eggers' or Peters' test, for quantifying publication bias, was not performed due to low statistical power (i.e., the number of studies in two subgroups was less than 10), and that is also the most likely reason for asymmetrical funnel test. Larger studies, which are likely to have more rigorous methodology, showed larger effect sizes. However, it is plausible that some smaller studies might have done things differently (methodology) and that could have made their results stronger. The heterogeneity within the countries may be present due to the influence of diverse variables, including socioeconomic status, lifestyle factors, cultural practices, and hospital IPC policies. It is noteworthy to consider that the presence of high levels of heterogeneity may indicate large variation in the seroprevalence of pre-existing IgG antibodies in HCWs across diverse geographic regions, age cohorts, genders, and community transmission. However, we did not find detailed background information on covariates (e.g., diverse geographic regions, age cohorts, and gender) in the literature for sensitivity analyses from majority of the selected studies. To address this, we used a random-effects model, which assumes that the effects being assessed in the selected studies are not all identical rather follow some distribution with centre of distribution describing the average of the effects and the width describing the degree of heterogenicity.

Supplementary Table 1: Characteristics of healthcare workers by recent SARS-CoV-2 infection in Western Norway over 3 periods in 2020

Variable	1st Period 1 (Mar – May), n = 1,159			2nd Period (Jun – Sep), n = 533			Period 3 (Oct – Dec), n = 604		
	**Negative, N = 1,093[1]**	**Positive, N = 66[1]**	**p-value[2]**	**Negative, N = 525[1]**	**New positive, N = 8[1]**	**p-value[2]**	**Negative, N = 551[1]**	**New positive, N = 53[1]**	**p-value[2]**
**Age (y):** Median (IQR)	40 (30-51)	42 (30-50)	0.9		32 (31-47)	0.4	40 (31-51)	41 (32-49)	0.9
**Sex: Female**	873 / 1,087 (80%)	57 / 66 (86%)	0.3	440 / 523 (84%)	7 / 8 (88%)	>0.9	430 / 541 (79%)	36 / 51 (71%)	0.2
**Study site: Stavanger**	327 / 1,093 (30%)	29 / 66 (44%)	**0.024**	268 / 525 (51%)	2 / 8 (25%)	0.3	194 / 551 (35%)	2 / 53 (3.8%)	**<0.001**
**Occupation**			0.3			0.8			0.5
Physician	253 / 1,089 (23%)	12 / 66 (18%)		90 / 523 (17%)	1 / 7 (14%)		139 / 540 (26%)	5 / 12 (42%)	
Nurse	469 / 1,089 (43%)	35 / 66 (53%)		241 / 523 (46%)	4 / 7 (57%)		229 / 540 (42%)	4 / 12 (33%)	
Others	367 / 1,089 (34%)	19 / 66 (29%)		192 / 523 (37%)	2 / 7 (29%)		172 / 540 (32%)	3 / 12 (25%)	
**Occupational exposure[3]**	642 / 1,093 (59%)	42 / 66 (64%)	0.5	261 / 525 (50%)	5 / 8 (62%)	0.7	338 / 551 (61%)	50 / 53 (94%)	**<0.001**
**Household exposure**	88 / 1,089 (8.1%)	17 / 65 (26%)	**<0.001**	58 / 524 (11%)	0 / 8 (0%)	0.7	61 / 540 (11%)	2 / 12 (17%)	>0.9
**International travel**	65 / 894 (7.3%)	6 / 60 (10%)	0.2	35 / 495 (7.1%)	3 / 8 (38%)	**0.005**	43 / 536 (8.0%)	2 / 12 (17%)	0.6
**Respiratory symptoms**	156 / 1,037 (15.1%)	25 / 64 (39%)	**<0.001**	78 / 524 (15%)	3 / 8 (38%)	0.2	153 / 539 (28%)	9 / 12 (75%)	**0.001**
**Fever**	122 / 1,049 (12%)	23 / 61 (38%)	**<0.001**	22 / 77 (29%)	2 / 3 (67%)	0.4	48 / 430 (11%)	6 / 11 (55%)	**<0.001**
**Cough**	85 / 165 (52%)	9 / 16 (56%)	>0.9	39 / 77 (51%)	1 / 3 (33%)	>0.9	96 / 430 (22%)	3 / 11 (27%)	>0.9
**Dyspnoea**	92 / 1,048 (8.8%)	15 / 61 (25%)	**<0.001**	15 / 75 (20%)	1 / 3 (33%)	>0.9	25 / 430 (5.8%)	2 / 11 (18%)	0.3
**Comorbidity[4]**	95 / 1,085 (8.8%)	5 / 62 (8.1%)	>0.9	47 / 524 (9.0%)	0 / 8 (0%)	0.8	52 / 541 (9.6%)	2 / 12 (17%)	0.7

[1]Data are number (%) unless otherwise specified. Infection status was determined by SARS-CoV-2 RT-PCR and/or serology testing. The 2nd and 3rd study period excluded individuals who were SARS-CoV-2 positive in the previous periods

[2]Kruskal-Wallis rank sum test; Pearson's Chi-squared test. Bold values are statistically significant values with $P < .05$.

Abbreviations: y, years; IQR, interquartile range.

[3]High-risk occupational group that tested and treated COVID-19 patients includes ambulances services; emergency, infectious diseases, anaesthesia, and intensive care unit departments at Haukeland University Hospital, Haraldsplass Deaconess Hospital, Bergen Municipality Emergency Room, and Stavanger University Hospital. Maternity ward of women's clinic at SUS was also deemed to be high-risk due to outbreak. Low-risk group that did not treat COVID-19 patients includes other clinical departments and laboratories. Low-risk group was reference;

[4]Comorbidities include hypertension, diabetes and heart disease.

Supplementary Table 2: SARS-CoV-2 infection rates and associated variables among healthcare workers in Western Norway in 2020

Variable	***Infection rate, mean (95% CI)****	***Adjusted odds ratio (95% CI)***	***Coef. (Std Error)***	***p-value****
**Age**	-	1.0 (1.0-1.1)	0.005 (0.02)	0.8
**Sex**				0.071
Female	7% (5-48%)	0.4 (0.1-1.1)	-0.98 (0.54)	
Male	17% (2-21%)	Ref.	Ref.	
**Occupational exposure to SARS-CoV-2**				0.4
High	14% (5-34%)	1.6 (0.5-4.7)	0.45 (0.56)	
Low	9% (2-32%)	Ref.	Ref.	
**Household exposure to SARS-CoV-2**				**0.001**
Exposed	28% (9-61%)	8.8 (2.4-32.1)	2.17 (0.66)	
Not exposed	4% (1-15%)	Ref.	Ref.	
**Travel history**				0.1
International	21% (6-55%)	2.9 (0.7-12.0)	1.05 (0.73)	
No	9% (2-29%)	Ref.	Ref.	
**Study site**				**0.030**
Bergen	22% (8-48%)	Ref.	Ref.	
Stavanger	6% (1-23%)	0.2 (0.1-0.9)	-1.58 (0.73)	
**Occupation**				0.4
Physician	9% (2-31%)	0.54 (0.13-2.16)	-0.62 (0.71)	
Nurse	10% (3-30%)	0.61 (0.19-1.94)	-0.49 (0.59)	
Others	16% (4-44%)	Ref.	Ref.	
**Comorbidity****				0.3
Present	7% (1-36%)	0.3 (0.1-2.3)	-1.16 (1.02)	
Absent	19% (9-36%)	Ref.	Ref.	

*Generalised additive mixed models including population level fixed effects and individual random effects (Infected, n = 127; non-infected, n = 1,087). Infection status determined by SARS-CoV-2 PCR and/or serology. Due to low community spread in Norway, we did find domestic travel as a risk factor. Bold values are statistically significant values with $P < .05$.
-Age was a continuous variable in the models (range 19-78 years). Infection rates increased with advancing age; however, age was not a main risk factor.
Abbreviations: CI, confidence intervals; Coef., regression coefficient; p, p-value; Ref., reference level or term;
**Comorbidities include hypertension, diabetes and heart diseases.

Supplementary Table 3: Infection prevention and control (IPC) policies in four major medical centres of Western Norway during 2020 and 2021

**SARS-CoV-2 IPC policies**	**Hospital 1: Haukeland University Hospital, Bergen, Hordaland**	**Hospital 2: Haraldsplass Hospital, Bergen, Hordaland**	**Hospital 3: Stavanger University Hospital, Rogaland**	**Community: Bergen Emergency Department, Hordaland**
Universal masking of HCWs	No	No	No	No
Facemasks, eye-shield, gown and gloves for HCWs at high-risk of occupational SARS-CoV-2 exposure	Pre-March 2020	Pre-March 2020	Pre-March 2020	Pre-March 2020
Shortages of PPE*	No	No	No	No
Surgical facemasks while managing COVID-19 patients**	Pre-March 2020	Pre-March 2020	Pre-March 2020	Pre-March 2020
Respirators (e.g., N95) for all aerosol-generating procedures (AGPs)	Pre-March 2020	Pre-March 2020	Pre-March 2020	Pre-March 2020
Universal eye protection for any patient encounters	No	No	No	No
Eye protection for confirmed or suspected cases	Pre-March 2020	Pre-March 2020	Pre-March 2020	Pre-March 2020
Universal testing for all inpatients/AGPs	No	No	No	No
Solicited history of COVID-19 risk or infection for all inpatients/AGPs	Pre-March 2020	Pre-March 2020	Pre-March 2020	Pre-March 2020

*Personal protective equipment (PPE) use was restricted and prioritised for HCWs at high-risk of SARS-CoV-2 infection, but there was no absolute shortage PPE use during contact with suspected and confirmed COVID-19 patients in these four centres, but there was reported shortage(2) of PPE for HCWs working in the community or at low-risk of exposure to SARS-CoV-2

** N95 respirators were not used for regular contacts with patients having COVID-19, however, it was used for all AGP procedures

Supplementary Table 4: Baseline characteristics of healthcare workers infected in 2020

Characteristics	Seronegative by 8 months, N = 15[1]	Seropositive by 8 months, N = 48[1]	p-value[2]
**Age (years): Median (IQR)**	40 (32-47)	44 (30-51)	0.7
**Sex**: Female	14 / 15 (93%)	40 / 48 (83%)	0.6
**Region**: Bergen, Vestland	12 / 15 (80%)	20 / 48 (42%)	**0.022**
**Anti-RBD SARS-CoV-2 antibodies[3]**	0.54 (0.48, 0.61)	1.70 (1.13, 2.14)	**<0.001**
**Anti-spike IgG SARS-CoV-2 antibodies[3]**	255 (195-351)	4,525 (1,319-9,775)	**<0.001**
**PCR**	0 / 2 (0%)	30 / 35 (86%)	**0.037**
**Comorbidity**	0 / 15 (0%)	4 / 44 (9.1%)	0.5
**Respiratory symptoms**	3 / 15 (20%)	8 / 47 (17%)	>0.9

[1]n / N (%)

[2]Kruskal-Wallis rank sum test; Pearson's Chi-squared test. Bold values are statistically significant values with P < .05

[3]Baseline antibodies, i.e., on day 1 of seropositive test result [Median (IQR, interquartile range)]

Supplementary Table 5: Characteristics of healthcare workers vaccinated against the coronavirus disease 2019 (COVID-19) in Western Norway

Variable	Jan-Feb 2021, N = 145			Mar-Jun 2021, N = 232		
	**Not infected, N = 137[1]**	**Infected, N = 8[1]**	**p-value[2]**	**Not infected, N = 186[1]**	**Infected, N = 46[1]**	**p-value[2]**
**Age (y):** Median (IQR)	39 (31-49)	50 (47-57)	**0.019**	42 (32-50)	45 (31-50)	0.6
**Sex: Female**	96 / 137 (70%)	6 / 8 (75%)	>0.9	136 / 186 (73%)	41 / 46 (89%)	**0.036**
**Occupation**			0.4			0.6
Other	18 / 137 (13%)	1 / 8 (12%)		34 / 186 (18%)	10 / 46 (22%)	
Physician	37 / 137 (27%)	4 / 8 (50%)		44 / 186 (24%)	8 / 46 (17%)	
Nurse	82 / 137 (60%)	3 / 8 (38%)		108 / 186 (58%)	28 / 46 (61%)	
**Vaccine type**			**<0.001**			**<0.001**
BNT162b2	137 / 137 (100%)	6 / 8 (75%)		159 / 186 (85%)	20 / 46 (43%)	
ChAdOx1-S and BNT162b2	0 / 137 (0%)	2 / 8 (25%)		27 / 186 (15%)	26 / 46 (57%)	
**Comorbidity[3]**	12 / 137 (8.8%)	2 / 8 (25%)	0.4	19 / 186 (10%)	3 / 43 (7.0%)	0.7

[1]n/N.
[2]Kruskal-Wallis rank sum test; Pearson's Chi-squared test. Bold values are statistically significant values with *P* < .05.
Infection status before vaccination was determined by SARS-CoV-2 PCR and/or 2-step orthogonal ELISA testing in 2020.
Abbreviations: y, years; IQR, interquartile range;
[3]Comorbidities include hypertension, diabetes and heart disease

Supplementary Table 6: Ongoing lessons from the 2019 coronavirus disease, COVID-19, pandemic: Protecting healthcare workers (Adapted from the Norwegian Institute of Public Health recommendations for use of personal protective equipment)

Risk category	Recommendations for the use of protective equipment
**Occupational exposure during contact with patients with**	
Any confirmed respiratory virus infection	Mask*, eye protection, gowns, gloves in well-ventilated spaces/buildings.<br>Isolation for the duration of the illness, often a minimum of 3-4 days after symptom onset.<br>An individual assessment of the need for prolonged isolation if symptoms persist and/or severe immunodeficiency and other risk factors.
Respiratory symptoms**	Measures are assessed based on symptoms and medical history.<br>Masks and eye protection for close contact with patients with respiratory symptoms***
Other patients**	Basic infection prevention routines****
**Household exposure or occupational exposure from colleagues**	
New-onset respiratory symptoms without a known agent/infectious agent	Symptomatic, regardless of vaccination status, must not go to work.<br>Return to work when well, negative RT-PCR test and fever-free for at least 24 hours.<br>Mask when in close contact with patients until they are symptom-free. They should strive to keep their distance from colleagues and use a mask when in close contact.
HCWs with any confirmed respiratory virus infection	Mask use when in close contact with patients, colleagues or others.

* Generally, protective equipment is only needed if you are closer than 2 metres to the patient with symptoms of any respiratory viral infection. Respirators (e.g., N95) for everyone staying in the same room where aerosol generating (AGP) procedures are carried out (3). Consider the use of a head cover (hair protection) in situations where there is a risk of direct soiling of the hair. Long hair should be securely tied back and off the neck. Due diligence should be used during removal of PPE to avoid contamination of clothing, skin and mucous membranes (including eyes).

** The infection regimen recommended for the current agent/disease must be implemented as soon as causative organism is identified.

*** Using a mask on the patient himself is an effective infection control measure if the patient's state of health allows it (e.g., in waiting rooms/common rooms, doctor's offices, outpatient clinics, during X-rays and during transport).

**** According to the basic infection control routines, the use of personal protective equipment must be assessed in every patient situation, based on the degree of risk of encountering body fluids or other contaminated objects.

Supplementary Table 7: Previously circulating variants of concerns (VOCs)

WHO label	Pango Lineage*	Earliest documented samples	Date of VOC designation	Earliest documented samples in Norway	Community spread
Alpha	B.1.1.7	United Kingdom, Sep-2020	18-Dec-2020	Oct - 2020	High
Beta	B.1.351	South Africa, May-2020	18-Dec-2020	Dec - 2020	Low
Gamma	P.1	Brazil, Nov-2020	11-Jan-2021	Mar - 2021	Very low
Delta**	B.1.617.2	India, Oct-2020	VOI: 4-Apr-2021<br>VOC: 11-May-2021	April - 2021	High

*Includes all descendent lineages
**HCWs were recruited in 2020 and followed up until June 2021.

Sources(4-6)

Supplementary Table 8: Advices from Norwegian Ministries about the Coronavirus disease (COVID-19)

Advices	
2020	
12 Mar	*National lockdown*
16 Mar	*Non-residents were banned from entering Norway*
19 Mar	*Residents prohibited from staying in cabins outside their home municipalities*
15 Jun	*Exceptions to the travel advice for Nordic countries*
15 Jul	*Non-essential travel exceptions for countries and regions that satisfy the Norwegian criteria for infection levels*
19 Aug	*Advice against non-essential travel to Austria, Greece, Ireland and the UK*
26 Aug	*Advice against non-essential travel to Germany and Liechtenstein, as well as to the regions of Kalmar and in Sweden*
23 Oct	*Immediate family members from outside the EU/EEA allowed to visit Norway (grandparents excluded)*
27 Oct	*New targeted measures (e.g., limiting number of guests/attendees; 5 at private, 50 at public, 600 at outdoor gatherings)*
05 Nov	*Advised to stay at home and have as little social contact as possible*
07 Nov	*Negative COVID-19 test required to enter Norway*
02 Dec	*Up to 10 guests allowed on the two days of Christmas at private social gathering*
14 Dec	*Removal of the requirements to stay in a quarantine hotel for persons with access to suitable accommodation*
21 Dec	*Banned direct flights from the United Kingdom, and registration requirement for all people entering Norway*
29 Dec	*New quarantine rules so more people get tested and to improve compliance with the quarantine rules*
2021	
02 Jan	*Mandatory testing for travellers to Norway and lifted ban on flights from the United Kingdom*
04 Jan	*Two weeks of stricter measures (e.g., work from home advice, avoiding guests at home & non-essential travel, digital teaching etc.)*
18 Jan	*Continuation of most national measures but easing of measures for children and young people*
23 Jan	*Stricter measures in ten municipalities after the UK COVID-19 mutation outbreak*
29 Jan	*Stricter rules on foreign nationals who seek entry to Norway (e.g., mandatory testing at the border and quarantine)*
20 Feb	*Stricter rules in relation to the use of quarantine hotels and applicated-based exemption from the entry restrictions.*
01 Mar	*Daily commuters from Sweden and Finland allowed to work in Norway under a strict testing and control regime*
10 Mar	*NIPH recommended ChAdOx1-S vaccine for those >65 years old and redistribution of vaccines to six districts with high rates of infection*
22 Mar	*Limited expansion of exemption scheme to include specialist construction/maintenance personnel*
25 Mar	*Stricter national measures (e.g., one-meter rule replaced two-meter rule, only 2 guests allowed at home, prohibited organized indoor activities with exception of elite athletes and fitness centres in resident's municipality, closed amusement parks, work from home advice)*

10 Apr	*Plans for gradual reopening*
21 May	*Changed the status of Iceland and several hospital districts in Finland from 'red' to 'yellow' on the quarantine on entry map*
24 May	*Travelers from the UK and countries in the EEA/Schengen area with a low rate of infection exempted from stay at a quarantine hotel*
04 Jun	*More people may quarantine in their own home*
07 Jun	*Shorter travel quarantine for protected/vaccinated people and children under the age of 12*
10 Jun	*Exemption from travel quarantine for people who are fully vaccinated or have recovered from COVID-19 during the past 6 months*
18 Jun	*Changed the status of Poland, Romania and certain hospital districts in Finland from 'red' to 'yellow' on the quarantine on entry map*
20 Jun	*Continued to reopen the society*

Source(7)

Supplementary Figure 1: Flow diagram of meta-analysis including current study. Search strategy: The electronic databases Google Scholar, MEDLINE, and Embase were searched using these keywords: COVID-19, COVID, coronavirus, Wuhan, 2019, SARS, SARS-CoV-2, coronavirus 2, 2019-ncov, SARS-2, health worker, health workforce, health professional, nurse, sero-epidemiologic studies, seroprevalence, antibodies, serologic tests, risk factors, immunoglobulin, and IgG. **Eligibility criteria:** Eligible studies met the following inclusion criteria: 1) published from January 1, 2020, to Dec 31, 2022, and 2) evaluated the association between occupational and/or household exposure to SARS-CoV-2 and risk of developing SARS-CoV-2 IgG specific antibodies. **Selection and screening of articles**: Original research articles published during COVID-19 pandemic until 31 December 2022 in the English language were searched for prospective and retrospective full text studies that reported quantitative data on association between SARS-CoV-2 IgG spike-specific antibodies in HCWs and occupational exposure (low or high-risk groups; or treating patients with or without PPE) or household exposure. Studies were excluded based on the title and abstract. Articles resulting from these searches and relevant references cited in those articles were reviewed. We accessed 4544 studies. After screening, 20 out of 125 studies were included in this review, including the current Norwegian cohort study [occupational(8-23) exposure to SARS-CoV-2 cases, no PPE use at work (9, 10, 17, 23-26), and household(9, 13, 19, 22, 26) exposure to SARS-CoV-2 cases]; excluding 105 studies: a. studies not reporting quantitative measures e.g., number of seropositive and negative HCWs based on occupational exposure, i.e., low or high risk groups; or treating patients with or without PPE or household exposure to patients with COVID-19, n = 66; b. heterogenous sample with mixed healthcare and non-healthcare workers, n = 37; and c. studies not available in English, n = 2). **Data extraction:** We extracted contextual data relevant to our study, including first author, country and timing of data collection.

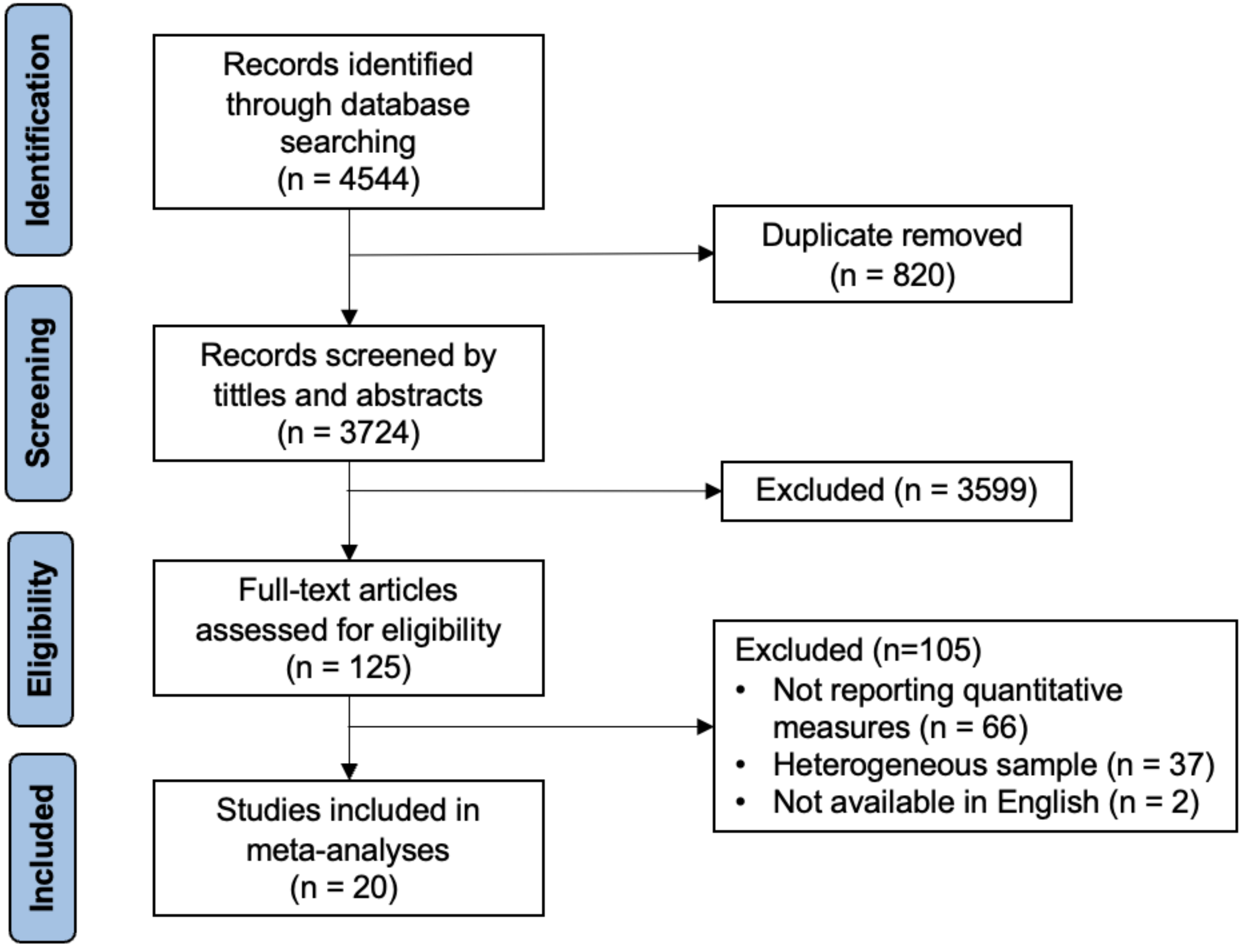
Identification
Screening
Eligibility
Included
Records identified through database searching (n = 4544)
Duplicate removed (n = 820)
Records screened by tittles and abstracts (n = 3724)
Excluded (n = 3599)
Full-text articles assessed for eligibility (n = 125)
Excluded (n=105)
• Not reporting quantitative measures (n = 66)
• Heterogeneous sample (n = 37)
• Not available in English (n = 2)
Studies included in meta-analyses (n = 20)

Supplementary Figure 2: Relationship and time course of infection status. Each row represents all test results for each healthcare worker with confirmed SARS-CoV-2 infection over the study period in 2020 (measured by SARS-CoV-2 anti-IgG spike antibodies and PCR test). Circles represent PCR and seronegative (dark green), PCR negative and seropositive (light firebrick), and PCR and seropositive (dark firebrick) at the time of sera collection. Squares represent previously PCR positive and seronegative (light cyan), previously PCR positive and seropositive (dark cyan). Diamonds represent PCR not tested and seronegative (light cornsilk), and PCR not tested and seropositive (dark cornsilk). Created in R with the ggplot2 package(27).

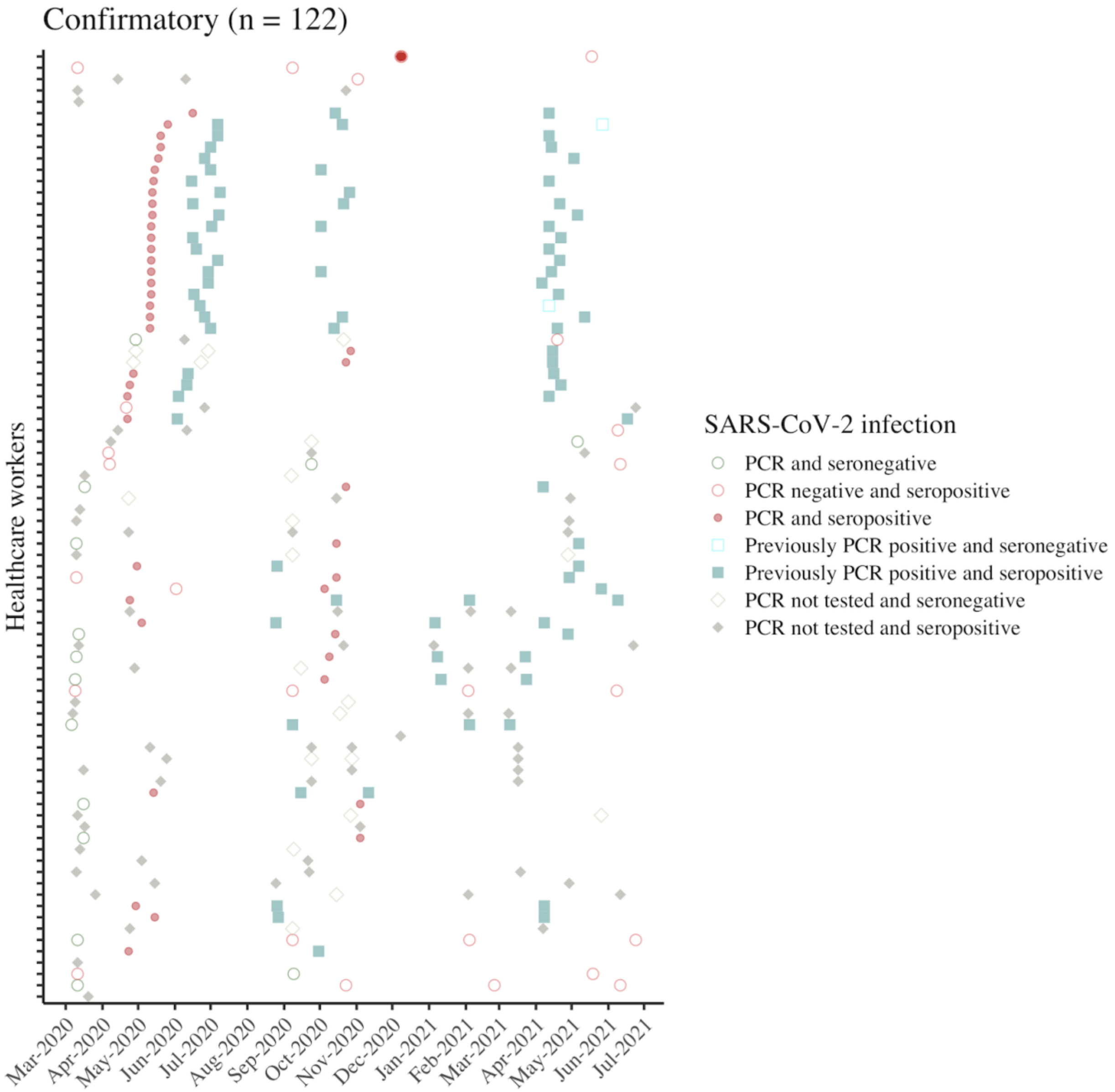
Confirmatory (n = 122)
Healthcare workers
Mar-2020
Apr-2020
May-2020
Jun-2020
Jul-2020
Aug-2020
Sep-2020
Oct-2020
Nov-2020
Dec-2020
Jan-2021
Feb-2021
Mar-2021
Apr-2021
May-2021
Jun-2021
Jul-2021
SARS-CoV-2 infection
PCR and seronegative
PCR negative and seropositive
PCR and seropositive
Previously PCR positive and seronegative
Previously PCR positive and seropositive
PCR not tested and seronegative
PCR not tested and seropositive

References

1. Balduzzi S RG, Schwarzer G. How to Perform a Meta-Analysis with R: A Practical Tutorial. *Evidence-Based Mental Health* (2019):153–60. doi: 10.1136/ebmental-2019-300117.
2. KS. Nsf Og Fagforbundet Ber Om Strakstiltak for Å Øke Tilgangen Til Nødvendig Smittevernsutstyr I Kommunene (2020). Available from: https://www.ks.no/informasjon-om-koronaviruset/helse-og-omsorg/ks-nsf-og-fagforbundet-ber-om-strakstiltak-for-a-oke-tilgangen-til-nodvendig-smittevernsutstyr-i-kommunene/
3. Norwegian Institute of Public Health. Bruk Av Personlig Beskyttelsesutstyr (2022) [updated 08.04.2022; cited 2022 October 24]. Available from: https://www.fhi.no/historisk-arkiv/covid-19/koronavirusveilederen-arkiv/personlig-beskyttelsesutstyr/.
4. World Health Organization. Tracking Sars-Cov-2 Variants (2022) [cited 2022 November 5]. Available from: https://www.who.int/activities/tracking-SARS-CoV-2-variants/.
5. Folkehelseinstituttet. Statistikk over Meldte Tilfeller Av Den Engelske Og Den Sørafrikanske Varianten Av Koronavirus (2021) [updated 08.03.2021; cited 2022 24 October]. Available from: https://www.fhi.no/sv/smittsomme-sykdommer/corona/statistikk-over-tilfeller-av-koronavirusvarianter/.
6. Folkehelseinstituttet. Covid-19 Ukerapport 15 - Uke 15 (2021) [updated 21 April, 2021; cited 2022 October 24]. Available from: https://www.fhi.no/contentassets/8a971e7b0a3c4a06bdbf381ab52e6157/vedlegg/alle-ukerapporter-2021/ukerapport-uke-15-12.04---18.04.21.pdf
7. Norwegian Government Security and Service Organisation (G.S.S.O.). Timeline: News from Norwegian Ministries About the Coronavirus Disease Covid-19 (2022) [cited 2022 October 24]. Available from: https://www.regjeringen.no/en/topics/koronavirus-covid-19/timeline-for-news-from-norwegian-ministries-about-the-coronavirus-disease-covid-19/id2692402/.
8. Doernberg SB, Holubar M, Jain V, Weng Y, Lu D, Bollyky JB, et al. Incidence and Prevalence of Covid-19 within a Healthcare Worker Cohort During the First Year of the Sars-Cov-2 Pandemic. *Clin Infect Dis* (2022). Epub 2022/03/13. doi: 10.1093/cid/ciac210.
9. Akinbami LJ, Chan PA, Vuong N, Sami S, Lewis D, Sheridan PE, et al. Severe Acute Respiratory Syndrome Coronavirus 2 Seropositivity among Healthcare Personnel in Hospitals and Nursing Homes, Rhode Island, USA, July-August 2020. *Emerg Infect Dis* (2021) 27(3):823-34. Epub 2021/02/25. doi: 10.3201/eid2703.204508.
10. Howard-Anderson JR, Adams C, Sherman AC, Dube WC, Smith TC, Edupuganti N, et al. Occupational Risk Factors for Severe Acute Respiratory Coronavirus Virus 2 (Sars-Cov-2) Infection among Healthcare Personnel: A Cross-Sectional Analysis of Subjects Enrolled in the Covid-19 Prevention in Emory Healthcare Personnel (Cope) Study. *Infect Control Hosp Epidemiol* (2022) 43(3):381-6. Epub 2021/02/10. doi: 10.1017/ice.2021.54.
11. Trieu MC, Bansal A, Madsen A, Zhou F, Sævik M, Vahokoski J, et al. Sars-Cov-2-Specific Neutralizing Antibody Responses in Norwegian Health Care Workers after the First Wave of Covid-19 Pandemic: A Prospective Cohort Study. *J Infect Dis* (2021) 223(4):589-99. Epub 2020/11/29. doi: 10.1093/infdis/jiaa737.

12. Moscola J, Sembajwe G, Jarrett M, Farber B, Chang T, McGinn T, et al. Prevalence of Sars-Cov-2 Antibodies in Health Care Personnel in the New York City Area. *JAMA* (2020). doi: 10.1001/jama.2020.14765.
13. Rosser JI, Roltgen K, Dymock M, Shepard J, Martin A, Hogan CA, et al. Severe Acute Respiratory Coronavirus Virus 2 (Sars-Cov-2) Seroprevalence in Healthcare Personnel in Northern California Early in the Coronavirus Disease 2019 (Covid-19) Pandemic. *Infect Control Hosp Epidemiol* (2021) 42(9):1053-9. Epub 2020/12/10. doi: 10.1017/ice.2020.1358.
14. Rudberg AS, Havervall S, Manberg A, Jernbom Falk A, Aguilera K, Ng H, et al. Sars-Cov-2 Exposure, Symptoms and Seroprevalence in Healthcare Workers in Sweden. *Nat Commun* (2020) 11(1):5064. Epub 2020/10/10. doi: 10.1038/s41467-020-18848-0.
15. Iversen K, Bundgaard H, Hasselbalch RB, Kristensen JH, Nielsen PB, Pries-Heje M, et al. Risk of Covid-19 in Health-Care Workers in Denmark: An Observational Cohort Study. *The Lancet Infectious Diseases* (2020). doi: 10.1016/S1473-3099(20)30589-2.
16. Piccoli L, Ferrari P, Piumatti G, Jovic S, Rodriguez BF, Mele F, et al. Risk Assessment and Seroprevalence of Sars-Cov-2 Infection in Healthcare Workers of Covid-19 and Non-Covid-19 Hospitals in Southern Switzerland. *Lancet Reg Health Eur* (2021) 1:100013. Epub 2021/06/27. doi: 10.1016/j.lanepe.2020.100013.
17. Galan MI, Velasco M, Casas ML, Goyanes MJ, Rodriguez-Caravaca G, Losa-Garcia JE, et al. Hospital-Wide Sars-Cov-2 Seroprevalence in Health Care Workers in a Spanish Teaching Hospital. *Enferm Infecc Microbiol Clin (Engl Ed)* (2020). Epub 2021/01/25. doi: 10.1016/j.eimc.2020.11.015.
18. Steensels D, Oris E, Coninx L, Nuyens D, Delforge ML, Vermeersch P, et al. Hospital-Wide Sars-Cov-2 Antibody Screening in 3056 Staff in a Tertiary Center in Belgium. *JAMA* (2020) 324(2):195-7. Epub 2020/06/17. doi: 10.1001/jama.2020.11160.
19. Wilkins JT, Gray EL, Wallia A, Hirschhorn LR, Zembower TR, Ho J, et al. Seroprevalence and Correlates of Sars-Cov-2 Antibodies in Health Care Workers in Chicago. *Open Forum Infect Dis* (2021) 8(1):ofaa582. Epub 2021/01/16. doi: 10.1093/ofid/ofaa582.
20. Blairon L, Mokrane S, Wilmet A, Dessilly G, Kabamba-Mukadi B, Beukinga I, et al. Large-Scale, Molecular and Serological Sars-Cov-2 Screening of Healthcare Workers in a 4-Site Public Hospital in Belgium after Covid-19 Outbreak. *J Infect* (2021) 82(1):159-98. Epub 2020/08/03. doi: 10.1016/j.jinf.2020.07.033.
21. Ludewick H, Hahn R, Italiano C, Pereira L, Fatovich D, Saxton J, et al. Covid-19 Serosurvey of Frontline Healthcare Workers in Western Australia. *J Epidemiol Glob Health* (2022). Epub 2022/09/22. doi: 10.1007/s44197-022-00065-1.
22. Kahlert CR, Persi R, Gusewell S, Egger T, Leal-Neto OB, Sumer J, et al. Non-Occupational and Occupational Factors Associated with Specific Sars-Cov-2 Antibodies among Hospital Workers - a Multicentre Cross-Sectional Study. *Clin Microbiol Infect* (2021) 27(9):1336-44. Epub 2021/05/22. doi: 10.1016/j.cmi.2021.05.014.
23. Ghosh S, Yadav AK, Rajmohan KS, Bhalla S, Sekhawat VS, Prashant J, et al. Seropositivity of Severe Acute Respiratory Syndrome Coronavirus 2 Infection among Healthcare Workers of the Armed Forces Medical Services, India: A Multicentric Study. *Med J Armed Forces India* (2021) 77(Suppl 2):S359-S65. Epub 2021/08/03. doi: 10.1016/j.mjafi.2021.03.020.

24. Mukwege D, Byabene AK, Akonkwa EM, Dahma H, Dauby N, Cikwanine Buhendwa JP, et al. High Sars-Cov-2 Seroprevalence in Healthcare Workers in Bukavu, Eastern Democratic Republic of Congo. *Am J Trop Med Hyg* (2021) 104(4):1526-30. Epub 2021/02/17. doi: 10.4269/ajtmh.20-1526.
25. Brehm TT, Schwinge D, Lampalzer S, Schlicker V, Kuchen J, Thompson M, et al. Seroprevalence of Sars-Cov-2 Antibodies among Hospital Workers in a German Tertiary Care Center: A Sequential Follow-up Study. *Int J Hyg Environ Health* (2021) 232:113671. Epub 2020/12/19. doi: 10.1016/j.ijheh.2020.113671.
26. Kohler PP, Kahlert CR, Sumer J, Flury D, Gusewell S, Leal-Neto OB, et al. Prevalence of Sars-Cov-2 Antibodies among Swiss Hospital Workers: Results of a Prospective Cohort Study. *Infect Control Hosp Epidemiol* (2021) 42(5):604-8. Epub 2020/10/09. doi: 10.1017/ice.2020.1244.
27. Wickham H. ggplot2: Elegant Graphics for Data Analysis: Springer-Verlag New York (2016). Available from: https://ggplot2.tidyverse.org.
